# Distinct *Leishmania* Species Infecting Wild Caviomorph Rodents (Rodentia: Hystricognathi) from Brazil

**DOI:** 10.1371/journal.pntd.0003389

**Published:** 2014-12-11

**Authors:** Renata Cássia-Pires, Mariana C. Boité, Paulo S. D'Andrea, Heitor M. Herrera, Elisa Cupolillo, Ana Maria Jansen, André Luiz R. Roque

**Affiliations:** 1 Laboratory of Trypanosomatid Biology, Oswaldo Cruz Institute, Rio de Janeiro, Rio de Janeiro, Brazil; 2 Laboratory of Leishmaniasis Research, Oswaldo Cruz Institute, Rio de Janeiro, Rio de Janeiro, Brazil; 3 Laboratory of Biology and Parasitology of Wild Reservoir Mammals, Oswaldo Cruz Institute, Rio de Janeiro, Rio de Janeiro, Brazil; 4 Dom Bosco Catholic University, Campo Grande, Mato Grosso do Sul, Brazil; Charité University Medicine Berlin, Germany

## Abstract

**Background:**

Caviomorph rodents, some of the oldest *Leishmania* spp. hosts, are widely dispersed in Brazil. Despite both experimental and field studies having suggested that these rodents are potential reservoirs of *Leishmania* parasites, not more than 88 specimens were analyzed in the few studies of natural infection. Our hypothesis was that caviomorph rodents are inserted in the transmission cycles of *Leishmania* in different regions, more so than is currently recognized.

**Methodology:**

We investigated the *Leishmania* infection in spleen fragments of 373 caviomorph rodents from 20 different species collected in five Brazilian biomes in a period of 13 years. PCR reactions targeting kDNA of *Leishmania* sp. were used to diagnose infection, while *Leishmania* species identification was performed by DNA sequencing of the amplified products obtained in the HSP70 (234) targeting. Serology by IFAT was performed on the available serum of these rodents.

**Principal findings:**

In 13 caviomorph rodents, DNA sequencing analyses allowed the identification of 4 species of the subgenus *L.* (*Viannia*): *L. shawi*, *L. guyanensis*, *L. naiffi*, and *L. braziliensis*; and 1 species of the subgenus *L.* (*Leishmania*): *L. infantum*. These include the description of parasite species in areas not previously included in their known distribution: *L. shawi* in *Thrichomys inermis* from Northeastern Brazil and *L. naiffi* in *T. fosteri* from Western Brazil. From the four other positive rodents, two were positive for HSP70 (234) targeting but did not generate sequences that enabled the species identification, and another two were positive only in kDNA targeting.

**Conclusions/Significance:**

The infection rate demonstrated by the serology (51.3%) points out that the natural *Leishmania* infection in caviomorph rodents is much higher than that observed in the molecular diagnosis (4.6%), highlighting that, in terms of the host species responsible for maintaining *Leishmania* species in the wild, our current knowledge represents only the “tip of the iceberg.”

## Author Summary

Leishmaniasis is a major public health problem expanding in Brazil and one of the reasons is that we still have poor knowledge of some aspects of the biology and epidemiology of *Leishmania* species, including the role of wild mammals. Caviomorph rodents, some of the oldest *Leishmania* spp. hosts, are widely dispersed in Brazil and reported as potential reservoirs of *Leishmania* parasites. Spleen fragments of 373 brazilian caviomorph rodents from 20 species were investigated for *Leishmania* infection. The molecular algorithm proposed to diagnose the infection associate the sensitivity of a molecular target with multiple copies with the specificity of another marker with discriminatory taxonomic ability between species. These demonstrated their usefulness in identifying most of the parasite species infecting the rodents, including the description of species in previously unknown hosts and in areas not previously included in their known distribution, such as *L. shawi* in *Thrichomys inermis* from Northeastern Brazil and *L. naiffi* in *T. fosteri* from Western Brazil. Although the percent of infection by molecular diagnosis was 4.6%, the serology demonstrated that about 51% of them had been exposed to *Leishmania* parasites pointing that caviomorph rodents are inserted in enzootic cycles of *Leishmania*, to a higher extent than currently recognized.

## Introduction

More than 30 species within the *Leishmania* genus (Trypanosomatidae, Trypanosomatida) are recognized, including both extremely specific as well as generalist species transmitted by a variety of Phlebotomine vectors worldwide [1]–[2] In fact, several *Leishmania* species are multi-host parasites that infect mammal species of different orders, including humans [3]–[4]. The diversity of *Leishmania* species, mammal hosts and environments in which the transmission occurs reveals a complex system. Leishmaniasis has an important impact on public health since it results in a spectrum of debilitating diseases, which can progress to very severe, even fatal cases. In Brazil, human cutaneous leishmaniasis is associated with at least six different species of the subgenus *Leishmania* (*Viannia*) besides *Leishmania* (*Leishmania*) *amazonensis*, while the visceral form is exclusively associated with *Leishmania* (*L.*) *infantum* [5]–[7].

Although usually interpreted as such, the description of *Leishmania* DNA in a given mammal species is not sufficient to consider this species a reservoir host. Reservoir is better defined not as a single species, but as an assemblage of species responsible for the long lasting maintenance of a parasite in a given environment [8]. In reservoir systems, each species of mammal host plays a unique role in the maintenance of the parasite, which means that these systems should always be considered in a restricted spatio-temporal scale, particular to each site and each moment [9]. Failures in interrupting human transmission and preventing new epidemics are probably related to the lack of knowledge of various aspects of the natural transmission cycles of these parasites. In this sense, the involvement of synanthropic hosts, such as caviomorph rodents and their potential to act as reservoirs cannot be ignored.

Although the diagnosis of *Leishmania* sp. infection in mammal tissues through molecular assays has been conducted by several groups, the identification of the *Leishmania* species is still a great challenge [10]–[12]. The parasite isolation in wild mammals is complex to due to the difficulties in performing aseptic culture during field expeditions. Besides, the usually observed low parasite load and irregular distribution of parasites among host tissues impair even more the isolation efficiency [13]–[17]. To overcome such limitations, molecular approaches have been developed and applied aiming to detect and identify *Leishmania* species directly in biological samples. The advantage of molecular approaches based on PCR is that they combine high sensitivity for direct detection of the infecting parasites in various human, animal and sand fly tissues, with species specificity [18]. The PCR followed by either Restriction Fragment Polymorphism (RFLP) or DNA sequencing of distinct targets have already been employed in biological samples. Not all of them are, however, useful for identification at the species level. Molecular targets directed to the conserved region of the kinetoplast minicircles of *Leishmania* are the most used for diagnosis due to their sensitivity (which is related to the high number of copies in a single parasite (∼10.000) but detects only the subgenerum level. The PCR-RFLP of the internal transcribed spacer 1 (ITS1) is the assay commonly used for direct detection and identification of *Leishmania* species in the Old World, but for Brazilian species presents a lower resolution. Among the targets that result in intraspecific variability, the *Heat Shock Protein* of 70 kDa is encoded by a polymorphic gene which has regions allowing diagnosis of the *Leishmania* species circulating in Brazil [19].

Rodents comprise more than two hundred species distributed in many different habitats. In fact, in nature, we found semi-aquatic, terrestrial and semi-fossorial species [20]. This trait is probably the main reason explaining their exposure to the different transmission cycles of several species of *Leishmania* in the wild [8]. The South American rodents are divided into two sub orders (Sciurognathi and Hystricognathi). The first Caviomorph rodents (Rodentia: Hystricognathi) arrived in the Americas about 35mya (million years ago), much earlier than other rodent groups [21]. Along with the first caviomorph migrants, during the Oligocene, it has already been proposed that new species of the *Leishmania* (*Leishmania*) subgenus may have arrived and subsequently diversified in the native mammal fauna [22].

Nowadays, caviomorph rodents are widely distributed in Brazil and contribute significantly to the animal biomass of these habitats. They are also found in peridomestic environments that report the circulation of *Leishmania* parasites among other species of mammals, humans and/or dogs [23]–[26]. To date in Brazil, there are few studies of natural *Leishmania* infection in caviomorph rodents and not more than 88 specimens were analyzed [27]–[29]. Furthermore, experimental studies pointed to the putative role of two caviomorph species as *Leishmania* reservoirs: *Thrichomys laurentius* infected by *L. infantum* and *L. braziliensis* [17] and *Proechimys semispinosus* infected with *L. infantum* [30]. In this sense, the analysis of different caviomorph rodent species from different regions and collected at different periods of time, may define the role of these taxa in the reservoir host system of *Leishmania* sp. In the present study we investigate the infection by *Leishmania* spp. in 373 caviomorph rodents from 20 different species and discuss the role of this group in the maintenance of the transmission cycles of *Leishmania* spp. in different regions of Brazil.

## Materials and Methods

### Animal samples

We evaluated spleen fragments from caviomorph rodents (n = 373) collected in five out six Brazilian biomes between 1999 and 2012 (S1 Table). These animals were captured using Tomahawk and Shermann “live-traps” during studies conducted by our laboratory [31]–[33]. In these studies, whenever the euthanasia of animals were realized, usually for taxonomic identification and/or diagnosis of parasite's infection, spleen fragments were collected in plastic tubes containing ethanol and stored at −20°C.

### Molecular diagnosis

All tissue fragments were re-hydrated with Nuclease-free water and the DNA extraction was performed using the Wizard Genomic DNA Purification Kit (Promega, Madison, USA) according to the manufacturer's recommendations. Positive and negative controls were derived from fragments of spleen and liver from infected (*Leishmania braziliensis*–IOC-L2483) and non-infected hamsters provided by the animal facilities of the Oswaldo Cruz Foundation. PCR were conducted using the pureTaq Ready-To-Go PCR beads (Amersham Biosciences, Buckinghamshire, UK) and primers directed to the conserved region of the *Leishmania* -kDNA minicircle, as follows: forward: 5′ – GGGAGGGGCGTTCTGCGAA-3′ and reverse: 5′ – GGCCCACTAT ATTACACCAACCCC – 3′ [17]. The PCR products were visualized after electrophoresis on 8% polyacrylamide gel and silver staining using a specific kit (DNA Silver Staining, GE Healthcare). Positive samples were submitted to a new PCR directed to a fragment of 234 bp of the *hsp70* gene, using the following primers (5′ - GGA CGA GAT CGA GCG CAT GGT - 3′) and (5′- TCC TTC GAC GCC TCC TGG TTG - 3′) [19]. The PCR amplifications were performed in a final volume of 50 µL containing 5 µL of DNA, 0.2 pmol of each primer, 0.2 µM dNTPs, 1.5 mM MgCl_2_ and 1 U GoTaq®DNA polymerase (Promega). The PCR assays used the following amplification cycle: 94°C for 5 min followed by 30 cycles of 94°C for 30 sec, 63°C for 1 min and 72°C for 1 min and a final extension at 72°C for 10 min. To increase the number of DNA copies, products obtained in the first reaction were submitted to a second PCR with the same primers and conditions described.

The PCR products obtained for HSP70 (234) targeting and the products positive only in kDNA targeting were purified using the Wizard SV Gel kit and PCR Clean-up System kit (Promega). The both products were sequenced with the same primers used for the PCR assay using the ABI PRISM BigDyeTerminator v3.1 Cycle Sequencing Kit. Sequencing was performed on an automated DNA sequencer (ABI PRISM_®BigDye_™ Terminator Cycle Sequencing) at the Genomic Platform – DNA Sequencing (PDTIS – FIOCRUZ). Consensus sequences were aligned and edited with the BioEdit Version v7.1.11 [34] and Phred/Phrap/Consed package Version: 0.020425.c [35] from two forward and two reverse strands. Sequences with Phred values below ten over their extent were discarded and only sequence segments with values above twenty were used for contig construction. Contigs from all samples were manually assembled and aligned in MEGA4 [36]. For that samples that not generated good-quality consensus sequence, the PCR reaction and the DNA sequencing was repeated two or more times until we obtain sequence segments equal to the previously established criteria. Species identification was performed by similarity analysis obtained by Alignment Search Tool (BLAST) algorithm hosted by NCBI, National Institute of Health, USA (http://www.ncbi.nlm.nih.gov), against sequences available on GeneBank after calculation of the statistical significance of matches. Besides, we also compared our sequences with a panel of sequences for HSP70 (234) gene obtained from representative strains of different *Leishmania* species circulating in Brazil available in the *Leishmania* collection of the Oswaldo Cruz Institute/CLIOC (Table 1). This table was allowed the identification of the positions of the polymorphic site that differentiates the parasite species.

**Table 1:** Main polymorphisms along the HSP70 (234) sequences that allow the distinction of *Leishmania* species from reference strains.

*Leishmania* Species	Position of Polymorphyc Site																																		
	19	22	23	24	35	36	39	45	47	48	58	70	78	79	113	116	117	122	145	148	150	156	158	160	161	163	168	170	171	174	181	182	183	185	201
*L. (V) braziliensis*	T	G	T	C	C	A	C	A	A	T	G	G	G	T	G	G	C	A	G	G	G	G	A	C	G	G	A	T	C	C	C	A	C	G	A
*L. (V) shawi*	.	.	.	.	.	.	.	.	.	.	.	.	.	.	A	.	.	.	.	.	.	.	G	.	.	.	.	.	.	.	.	G	.	.	.
*L. (V) guyanenis*	.	.	.	.	.	.	.	.	.	.	.	.	.	.	A	.	.	.	.	.	.	.	.	.	.	.	.	.	.	.	.	.	.	.	.
*L. (V) naiffi*	.	.	.	.	.	.	.	.	.	.	.	.	.	C	.	.	.	.	.	.	.	.	.	.	A	.	C	.	.	.	.	G	.	.	.
*L. (V) lainsoni*	.	.	.	.	.	.	.	.	.	.	.	.	C	.	A	T	.	.	.	.	.	A	G	.	.	.	.	.	.	.	.	.	.	.	.
*L. (L) infantum*	C	.	A	T	G	C	A	G	G	C	C	A	.	C	C	.	G	T	C	T	.	A	G	.	A	.	.	A	A	A	.	G	T	A	G
*L. (L) amazonensis*	C	A	A	T	G	C	A	.	G	C	C	A	.	.	C	.	G	T	C	C	C	A	T	G	A	A	.	A	A	A	G	G	.	.	G

### Serological diagnosis

The immunofluorescence assay (IFAT) was performed on 130 available serum samples from the evaluated rodents, as described by Camargo (1966) [37]. A mixture of promastigotes from cultures of *L.* (*V.*) *braziliensis* (IOC/L566) and *L.* (*L.*) *infantum* (IOC/L579) obtained from CLIOC was used as antigen. In order to detect possible cross-reaction with *Trypanosoma cruzi*, sera were also tested using reference strains of *T. cruzi* (TcI M000/BR/1974/F and TcII MHOM/BR/1950/Y). The reactions were conducted using an *in-house* intermediary antibody anti-*Thrichomys* serum produced in rabbits [38]. The reaction was visualized using a commercial anti-rabbit IgG-FITC (Sigma-Aldrich, St. Louis, USA). Rodents that displayed serological titers 1/10 and 1/20 for *Leishmania* spp. infection were considered positive only if these titers were equal or higher than the titers observed in the same rodent for *T. cruzi* infection. Rodents that displayed *Leishmania* serological titers equal or higher than 1∶40 were considered positive independent of *T. cruzi* results.

### Ethics statement

All the procedures carried out with these animals were authorized by the Brazilian Institute of Environment and Renewable Natural Resources (IBAMA) and followed protocols approved by the Ethics Committee of Animal Use Fiocruz (P0007-99, P0179-03; P0292/06; L0015-07).

## Results

**Figure 1:**
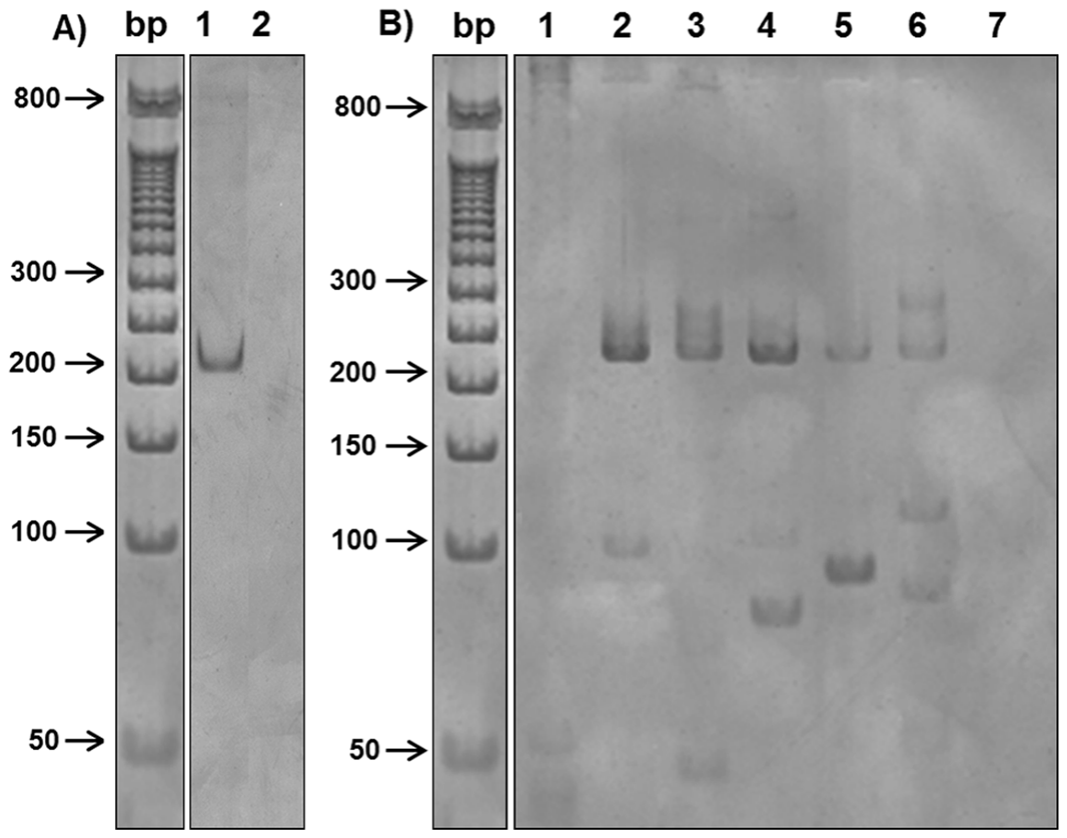
Illustrative representation of HSP 70 (234) amplification for *Leishmania* sp. before and after re-amplification of the PCR products. (A) PCR products of first amplification of HSP 70 (234) targeting analyzed by electrophoresis polyacrylamide gel stained with silver. Lanes: bp. molecular-weight marker (50 bp DNA ladder); 1. Infected *Thrichomys laurentius* from São Raimundo Nonato/PI; 2. Negative control of PCR reaction. (B) PCR products of re-amplification of the product obtained in the first PCR reaction. Lanes: bp. molecular-weight marker (50 bp DNA ladder); 1. Negative *Thrichomys fosteri* from Corumbá/MS (Positive only in kDNA); 2–5. Infected *T. laurentius* from São Raimundo Nonato/PI; 6. Infected *T. fosteri* from Corumbá/MS; 7. Negative control of PCR after re-amplification.

**Figure 2:**
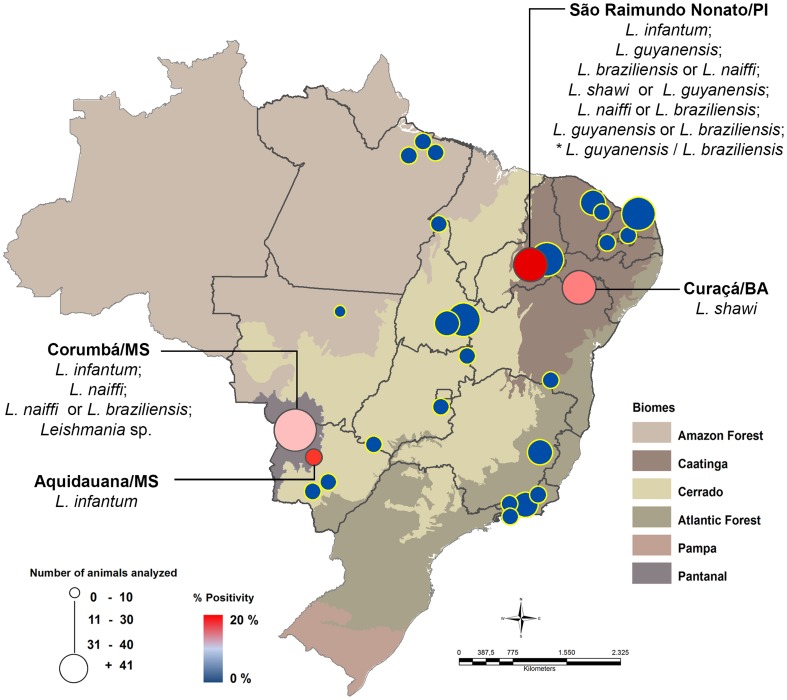
Map of the distribution of *Leishmania* species infecting caviomorph rodents in Brazil. Blue markers indicate municipalities where caviomorph rodents were collected, but were all negative in the molecular assay. Red markers indicate municipalities with animals positive for *Leishmania* infection. * Sample with high value between distinct species of *Leishmania* was considered as a result of hybrid or mixed infection.

We found 17 caviomorph rodents (4.6%) positive in the PCR directed to *Leishmania* sp. kDNA. From these, 15 samples were also positive when tested for HSP70 (234) primers, two directly after the PCR and 13 only after the re-amplification of the product obtained in the first PCR reaction (Fig. 1). DNA sequencing analyses allowed the identification of −4 species of the subgenus *Leishmania* (*Viannia*) and −1 species of the subgenus *Leishmania* (*Leishmania*) in samples of 13 caviomorph rodents (Fig. 2, Table 2). In five situations, the DNA sequence analysis revealed identity values above 95% for more than one *Leishmania* species and we opted not to define one of them as the etiological agent. A single sample (one *Thrichomys laurentius* from Piauí) displayed different DNA sequences that presented high values of similarity with distinct species of *Leishmania* in two consecutive reactions. This was considered as result of a hybrid population or mixed infection. *Leishmania* infection was observed in five caviomorph rodent species captured in two municipalities belonging to Pantanal and in two belonging to Caatinga biomes (Fig. 2). In the Pantanal, 7 animals were positive for *Leishmania* spp and the identification of the *Leishmania* species was possible in 4 of them: one *T. fosteri* captured in Corumbá, was found infected by *L.* (*V*). *naiffi* while *L.* (*L*). *infantum* was found infecting one *Dasyprocta azarae* and two *Clyomys laticeps* in Aquidauna and Corumbá, respectively. Also in Corumbá, we were unable to identify the *Leishmania* species infecting one *T. fosteri* because the analysis of the parasite DNA sequence revealed similarity with two *Leishmania* species (*L.* (*V*). *naiffi* and *L.* (*V*). *braziliensis*).

**Table 2:** Infected caviomorph rodents and their respective *Leishmania* species identified by the analysis of similarity between the DNA sequences from the PCR products targeting HSP70 (234) and available sequences from GeneBank.

Locality	Positive mammal	*Leishmania* identification	Max Score	Identity (%)
**Bahia**	*Thrichomys inermis*	*Leishmania* (*V*). *shawi*	422	99
**Mato Grosso do Sul**	*Thrichomys fosteri*	*Leishmaia* (*V*). *naiffi*	390	99
	*Dasyprocta azarae*	*Leishmania* (*L*). *infantum*	185	88
	*Clyomys laticeps*	*Leishmania* (*L*). *infantum*	427	99
	*Clyomys laticeps*	*Leishmania* (*L*). *infantum*	361	93
	*Thrichomys fosteri*	*Leishmaia* (*V*). *naiffi* **OR**	435	100
		*Leishmania (V). braziliensis*	424	99
**Piauí**	*Thrichomys laurentius*	*Leishmania* (*V*). *guyanensis*	433	100
	*Thrichomys laurentius*	*Leishmania* (*L*). *infantum*	303	97
	*Thrichomys laurentius*	*Leishmania* (*V*). *braziliensis* **OR**	422	99
		*Leishmaia* (*V*). *naiffi*	422	99
	*Thrichomys laurentius*	*Leishmania* (*V*). s*hawi* **OR**	390	95
		*Leishmania* (*V*). *guyanensis*	385	95
	*Thrichomys laurentius*	*Leishmaia* (*V*). *naiffi* **OR**	435	100
		*Leishmania* (*V*). *braziliensis*	424	99
	*Thrichomys laurentius*	*Leishmaia* (*V*). *naiffi* **OR**	422	99
		*Leishmania* (*V*). *braziliensis*	411	98
	*Thrichomys laurentius* *	*Leishmania* (*V*). *guyanensis*	416	100
		*Leishmania* (*V*). *braziliensis*	416	99

* Sample with high value between distinct species of *Leishmania* was considered as a result of hybrid or mixed infection.

From Caatinga biome, in the municipality of Curacá/Bahia, one *Thrichomys inermis* was found infected by *L.* (*V*). *shawi*. In São Raimundo Nonato/Piauí, two *T. laurentius* were found infected, respectively, by *L.* (*V*). *guyanensis* and *L.* (*L.*) *infantum*. In four *T. laurentius*, the identification of the *Leishmania* species was not possible because the parasite DNA sequencing analyses showed high similarity for two *Leishmania* species (Table 2). The specific diagnosis in the seventh *T. laurentius* was only possible after two attempts and showed the presence of two *Leishmania* species, *L.* (*V*). *braziliensis* and *L.* (*V*). *guyanensis* (S2 Table). This was interpreted as the result of a mixed infection and/or a possible hybrid of them.

The prevalence of antibodies against *Leishmania* sp. in IFAT was 51.3% (59/115). In 15 samples the results were inconclusive and were therefore not included in the final analysis. Among the samples analyzed (S1 Table), 56 animals were positive in IFAT but negative by PCR. From these, the majority of them (n = 34) displayed titers equal or higher than 1∶20 in the IFAT. Of the six animals positive for *Leishmania* kDNA, three were also positive by IFAT. Other 53 rodents were negative for both tests.

## Discussion

A clear expansion of leishmaniasis around the country has been observed in the last decades [23], [39]–[41]. From the different reasons already proposed to explain this expansion, a common point is the recognition that we still have poor knowledge of some aspects of the biology and epidemiology of *Leishmania* species, which, in the end, result in inefficient control strategies. The mammalian hosts of most *Leishmania* species are still poorly understood, which reinforces the need for studies that verify the distribution of these parasites in other mammalian taxa beyond those classically reported as reservoirs. Caviomorph rodents comprise an enormous group of species that exploit different habitats, and also include some species already domesticated by humans, such as the chinchilla and the guinea pig (*Cavia* spp.) [42].

Among the rodent species found infected in this study, only species from genus *Dazyprocta* and *Thrichomys* were already found infected by *Leishmania* spp. in Brazil. *Dasyprocta* sp., was found infected by *L. guyanensis* in Pará state [27] while *T. apereoides*, was founded infected by *L. braziliensis*, *L. guyanensis*, and *L. amazonensis* in Minas Gerais state [28]–[29]. Morevoer, other *Thrichomys* species were reported as potential reservoirs of trypanosome species in different regions of Brazil [33], [43]. We presented here the first description of *Leishmania* infection in *Clyomys laticeps*, a rodent species already found naturally infected by *T. evansi* and *T. cruzi* in Pantanal region” [33], [44].

*Thrichomys inermis* was the most abundant small mammal captured during the field expedition in Curaçá/Bahia, which points to its importance as a potential reservoir host. We described here for the first time, *L. shawi* in rodents, as well as this *Leishmania* species outside the Amazon region. Up to know, *L. shawi* is known to be transmitted only by *Lutzomyia whitmani* associated to arboreal and/or scansorial mammals (primates, sloths and coatis) suggesting a transmission cycle restricted to this forest strata [7], [45]. In a first view, our findings led to the suggestion that this transmission could also occur near the ground, since *T. inermis* is a terrestrial rodent that only rarely explores the understory strata [20]. However, we recently revealed through camera traps another species of this genus (*T. fosteri*) invading a coati nest about 12 meters high in the Pantanal region (Guilherme Mourão, personal communication). This finding reflects the lack of knowledge about the biology of some of the most widely distributed mammal species and the risk of incorrectly interpreting the parasite transmission cycle based on misconceptions of the biology of their mammal hosts and/or vectors.

American visceral leishmaniasis (AVL) is widely distributed in the state of Mato Grosso do Sul. Currently, the Aquidauana municipality displays an increasing number of human and canine cases in the urban area [46] and here we report for the first time the presence of *L. infantum* in sylvatic areas of this municipality. It is worth mentioning that although *Dazypropcta azarae* is a wild rodent species, these animals are frequently found in urban environments such as parks and woodlands. In these localities, these animals have no natural predators and are usually provided with food by local visitors, resulting in propitious conditions for reproduction. The presence of synanthropic *L. infantum* hosts in areas of intense transmission is a factor usually not considered in the epidemiology of VL. Nevertheless, we can not rule out the participation of other wild hosts in this transmission cycle, which is a complicating factor for the success of control strategies.

Also in the Pantanal region, the Corumbá municipality is endemic for AVL, recording dog's and human's cases [47]–[48]. In this municipality, there were conducted four expeditions between 2003 and 2009, and *Leishmania* infected rodents were found in all of them. The captures occurred in two farms that present different land uses. The Alegria Farm has livestock activities whereas the Nhumirim Farm is the center for scientific studies and presents large conservation areas. Two *Clyomys laticeps*, each one captured in one farm were found infected by *L. infantum*. This is especially important in the Alegria Farm where the interaction between wild and domestic mammals is common. All other infected rodents were captured in the Nhumirim Farm, pointing out that richness of biodiversity, as encountered in preserved areas, also reflect parasite diversity. At least one *Thrichomys fosteri* were found infected by *Leishmania naiffi* and this represents the first description of *Leishmania naiffi* in rodents and also of this *Leishmania* species outside the Amazon region [49]–[50]. Although we found no reports of the known *L. naiffi* vectors in the Mato Grosso do Sul state, their presence cannot be discarded or, alternatively, this transmission is being maintained by other phlebotomine species [51]. Another *T. fosteri* was founded infected by *L. naiffi* although the sequence of the amplified products revealed that *L. braziliensis* could not be discarded as etiologic agent of this infection. Indeed, these species differ in solely four nitrogenous bases at positions 79, 161, 168 e 182 when sequenced to target HSP70 (234) (Table 1).

Our data from São Raimundo Nonato/Piauí revealed the higher diversity of *Leishmania* species in a single host species, *Thrichomys laurentius*. This diversity could be observed in two consecutive years of expeditions. *T. laurentius* are a wild mammal species that has synanthropic habits and displayed high relative abundance in both expeditions. These rodents displayed high prevalence of infection that can potentially be considered a risk of infection not only for other wildlife populations, but also for domestic animals and men that expose themselves in areas of transmission. One *T. laurentius* was found infected by *L. infantum*. Indeed, the Piauí state is endemic for AVL with records of infection in marsupials, dogs and wild carnivores [52]–[55]. Another *T. laurentius* was found infected by *L. guyanensis*. This parasite species is commonly found in the Amazon region and known to be transmitted mainly by *Lutzomyia umbratilis*, a anthropophilic species that is generally found in understory strata [56].

Despite the difficulties, specific diagnosis is crucial to better understand the complex network of transmission of *Leishmania* species, which was achieved in the above mentioned situations. Ideally, studies that focus on the description of *Leishmania* reservoirs in a given area should be performed in long-termed studies. When not possible, punctual studies may also be quite informative, but it is imperative that this must to be analysed within a broad methodological approach that includes parasitological, serological and molecular diagnoses in different tissues. These factors will define the role of a given mammal host as a reservoir of *Leishmania* parasites. Nevertheless, this study design not always is possible due to inherent difficulties in working with wild mammals in the field. The molecular algorithm proposed here sought to combine the sensitivity of a molecular target and the specificity of DNA sequencing analysis of a locus. In fact, PCR targeted to the conserved region of kDNA has proved to be the most sensitive, but only allows the identification up to the subgenus level [15], [57]. Although less sensitive to diagnose the infection, the variable region of HSP 70 enabled the identification of the parasite species [19]. It is worth mentioning that in most cases positive reactions were only visualized after the re-amplification of the sequences obtained in the first reaction. This is the first time this approach is applied directly in tissue samples from wild mammals.

Also in São Raimundo Nonato, the analysis of the molecular identification of four animals resulted in similar sequences for more than one *Leishmania* species. One *T. laurentius* showed similar results for *L. shawi* and *L. guyanensis*, an expected result since both *Leishmania* species belong to the same complex and differ only in two bases, in HSP70 (234) sequence (Table 1). Other three *T. laurentius* showed similar results for *L. braziliensis* e *L. naiffi* (Table 2). We also obtained a divergent result after repeating the PCR reaction of one *T. laurentius* sample, which was interpreted as a result of a mixed infection or a hybrid between *L. braziliensis* and *L. guyanensis*. These five situations of inconclusive species identification reflect an inherent limitation of the employed methodology. On the other hand, it highlights how complex the identification of *Leishmania* species is (especially when sylvatic samples are used) and reflects how challenging is the establishment of a universal methodology, unique for diagnosis of the infection. In nature, the evolutionary success of *Leishmania* populations depends on their ability to multiply and be transmitted in different microenvironments (vertebrate and invertebrate hosts), which are both influenced by the habitat where they live. This process of evolution species is a dynamic, mutable and still poorly known phenomenon. Our attempt to describe the parasitic populations resulting from such complex interactions into discrete (not continuous) taxonomic units, although epidemiologically important, is always subject to findings like these. These should not be interpreted as an indicative to search for new discriminatory molecular markers, but instead as a natural report of the complexity that exists in *Leishmania* taxa.

The serological survey showed that 51.3% of the caviomorph rodents evaluated had become exposed to *Leishmania* parasites, and, therefore, are expected to be infected. However, we found only 4.6% of positive rodents through the molecular analysis of spleen fragments. *Leishmania* sp. has non-uniform distribution in tissues of vertebrate hosts, and may be present in other fragments of the spleen as well as in different tissues as skin and/or liver. The three animals that were positive for the molecular diagnosis, but negative for IFAT may have been caught in an initial phase of infection when there was not yet production of detectable IgG in serological tests. Another factor, although less known in wild hosts, may be an inability (permanent or temporary) of some individuals to produce detectable antibodies in serological assays. The rate of infection demonstrated by the serology points out that the natural *Leishmania* infection in caviomorph rodents is much higher than that observed in the molecular diagnosis. Our results based on PCR of spleen fragments and serology reflects only the “tip of the iceberg” highlighting that the knowledge about the epidemiology of different *Leishmania* species that infect caviomorph rodents is just at its beginning. The diversity of *Leishmania* species found infecting different caviomorph rodent species reflects the dynamism and complexity of the transmission cycles of these parasites in nature.

## Supporting Information

S1 Table Origin of the analyzed caviomorph rodents, which were collected in Brazil between 1999 and 2012.
(DOCX)

S2 Table Similarity levels observed by BLAST analysis among the DNA sequencing obtained for the target HSP70 (234) in the infected caviomorph rodents and available sequences from GeneBank with their respective accession numbers.
(DOCX)
