# Supplementary material for: Distinct Leishmania Species Infecting Wild Caviomorph Rodents (Rodentia: Hystricognathi) from Brazil
Source: PLoS Negl Trop Dis. 2014 Dec 11;8(12):e3389. doi: 10.1371/journal.pntd.0003389 (PMC4263410; doi:10.1371/journal.pntd.0003389)
Supplement: S1 Table — Origin of the analyzed caviomorph rodents, which were collected in Brazil between 1999 and 2012. (DOCX) [file pntd.0003389.s001.docx]

**Table S1: Origin of the analyzed caviomorph rodents, which were collected in Brazil between 1999 and 2012**

| **Biome/State** | **Municipalities** | **Species*** | **Number of analyzed animals** | | **Coordinates** | |
| --- | --- | --- | --- | --- | --- | --- |
|  |  |  | **PCR** | **IFAT** |  |  |
| **Amazon Forest** |  |  |  |  |  |  |
| Mato Grosso | Marcelândia | *Proechimys longicaudatus* | 3 |  | 11°02'46.57"S | 54°26'15.67"W |
| Pará | Belém | *Dasyprocta azarae* | 1 |  | 1°27'22.91"S | 48°29'23.41"W |
|  |  | *Proechimys* sp. | 1 |  |  |  |
|  | Cachoeira do Arari | *Proechimys goeldii* | 7 |  | 1°0'15.91"S | 48°57'26.49"W |
|  | Curralinho | *Proechimys cuvieri* | 4 | 4 | 1°48'47.50"S | 49°47'45.11"W |
| Tocantis | Araguatins | *Proechimys roberti* | 6 | 6 | 5°38'48.58"S | 48°7'23.78"W |
|  |  |  |  |  |  |  |
| **Atlantic Forest** |  |  |  |  |  |  |
| Minas Gerais | Capitão Andrade | *Cavia porcellus* | 1 |  | 19°4'29.41"S | 41°51'41.09"W |
|  |  | *Thrichomys apereoides* | 15 | 2 |  |  |
| Rio de Janeiro | Mangaratiba | *Trinomys* sp. | 1 |  | 22°57'33.84"S | 44°2'27.22"W |
|  | Piraí | *Sphiggurus villosus* | 1 |  | 22°37'19.37"S | 43°54'30.42"W |
|  | Sumidouro | *Sphiggurus villosus* | 2 |  | 22°2'54.61"S | 42°40'34.29"W |
|  | Teresópolis | *Trinomys dimidatus* | 12 |  | 22°24'58.87"S | 42°58'30.85"W |
|  |  | *Trinomys bonafidei* | 7 |  |  |  |
|  |  |  |  |  |  |  |
| **Caatinga** |  |  |  |  |  |  |
| Bahia | Curaçá | *Galea spixii* | 5 |  | 8°59'4.47"S | 39°53'58.81"W |
|  |  | *Thrichomys inermis* | 7 | 5 |  |  |
|  | Livramento | *Thrichomys* sp. | 3 |  |  |  |
|  | Tremedal | *Galea spixii* | 2 |  | 14°58'25.06"S | 41°24'51.12"W |
| Ceará | Jaguaruana | *Galea spixii* | 14 | 4 | 4°49'53.44"S | 37°46'51.60"W |
|  |  | *Thrichomys laurentius* | 21 | 5 |  |  |
|  | Redenção | *Thrichomys laurentius* | 11 | 8 | 4°12'57.14"S | 38°43'39.81"W |
|  | Russas | *Thrichomys laurentius* | 7 | 5 | 4°55'36.21"S | 37°58'19.52"W |
| Paraíba | Sousa | *Thrichomys laurentius* | 2 |  | 6°45'5.35"S | 38°13'51.82"W |
| Piauí | Coronel José Dias | *Thrichomys laurentius* | 32 |  | 8°48'50.27"S | 42°31'23.51"W |
|  | São Raimundo Nonato | *Thrichomys laurentius* | 26 | 17 | 9°0'44.69"S | 42°41'55.18"W |
| Rio Grande do Norte | Caicó | *Galea spixii* | 1 |  | 6°27'15.89"S | 37°6'24.21"W |
|  |  | *Thrichomys laurentius* | 3 |  |  |  |
|  |  |  |  |  |  |  |
| **Cerrado** |  |  |  |  |  |  |
| Goiás | Aporé | *Dasyprocta azarae* | 1 |  | 18°57'38.65"S | 51°55'23.49"W |
|  |  | *Proechimys roberti* | 1 |  |  |  |
|  | Luziânia | *Proechimys roberti* | 1 |  | 16°15'10.87"S | 47°57'0.04"W |
|  |  | *Thrichomys pachyurus* | 6 | 4 |  |  |
|  | São Domingos | *Thrichomys aff. apereoides* | 2 | 2 | 13°24'13.51"S | 46°19'8.29"W |
| Mato Grosso do Sul | Aquidauana | *Dasyprocta azarae* | 1 |  | 20°27'59.59"S | 55°47'12.43"W |
|  |  | *Thrichomys fosteri* | 5 |  |  |  |
|  | Maracaju | *Thrichomys fosteri* | 9 |  | 21°36'37.96"S | 55°10'3.96"W |
|  | Sidrolândia | *Thrichomys fosteri* | 1 |  | 20°55'48.77"S | 54°58'9.19"W |
| Tocantis | Dianópolis | *Proechimys roberti* | 8 | 2 | 11°37'26.44"S | 46°49'11.24"W |
|  |  | *Thrichomys inermis* | 4 |  |  |  |
|  | Novo Jardim | *Proechimys roberti* | 10 | 1 | 11°49'33.47"S | 46°37'56.91"W |
|  |  | *Thrichomys inermis* | 22 | 14 |  |  |
|  |  |  |  |  |  |  |
| **Pantanal** |  |  |  |  |  |  |
| Mato Grosso do Sul | Corumbá | *Clyomys laticeps* | 38 |  | 19°0'27.83"S | 57°39'3.74"W |
|  |  | *Dasyprocta azarae* | 3 |  |  |  |
|  |  | *Thrichomys fosteri* | 66 | 36 |  |  |
| **TOTAL** |  |  | **373** | **115** |  |  |

IFAT = Immunofluorescence assay

* Taxonomic identification was performed by morphological characteristics and karyological analyses, as described in Bonvicino *et al.,* 2002 (Bonvicino CR, Otazu IB, D’Andrea PS. Karyologic evidence of diversification of the genus *Thrichomys* (Rodentia, Echimyidae). Cyt Gen Res 97: 200–204).
