# Supplementary material for: Distinct Leishmania Species Infecting Wild Caviomorph Rodents (Rodentia: Hystricognathi) from Brazil
Source: PLoS Negl Trop Dis. 2014 Dec 11;8(12):e3389. doi: 10.1371/journal.pntd.0003389 (PMC4263410; doi:10.1371/journal.pntd.0003389)
Supplement: S2 Table — Similarity levels observed by BLAST analysis among the DNA sequencing obtained for the target HSP70 (234) in the infected caviomorph rodents and available sequences from GeneBank with their respective accession numbers. (DOCX) [file pntd.0003389.s002.docx]

**Table S2: Similarity levels observed by BLAST analysis among the DNA sequencing obtained for the target HSP70 (234) in the infected caviomorph rodents and available sequences from GeneBank with their respective accession numbers.**

| **Mammal host** | **Locality** | ***Leishmania* identification** | [**Max score**](http://www.ncbi.nlm.nih.gov/blast/Blast.cgi?CMD=Get&ALIGNMENTS=0&ALIGNMENT_VIEW=Pairwise&DATABASE_SORT=0&DESCRIPTIONS=100&DYNAMIC_FORMAT=on&FIRST_QUERY_NUM=0&FORMAT_OBJECT=Alignment&FORMAT_PAGE_TARGET=&FORMAT_TYPE=HTML&GET_SEQUENCE=yes&I_THRESH=&MASK_CHAR=2&MASK_COLOR=1&NCBI_GI=yes&NUM_OVERVIEW=100&OLD_BLAST=false&PAGE=MegaBlast&QUERY_INDEX=0&QUERY_NUMBER=0&RESULTS_PAGE_TARGET=&RID=EVX6WZ9X014&SHOW_LINKOUT=yes&SHOW_OVERVIEW=yes&STEP_NUMBER=&OLD_VIEW=false&DISPLAY_SORT=1&HSP_SORT=1) | [**Total score**](http://www.ncbi.nlm.nih.gov/blast/Blast.cgi?CMD=Get&ALIGNMENTS=0&ALIGNMENT_VIEW=Pairwise&DATABASE_SORT=0&DESCRIPTIONS=100&DYNAMIC_FORMAT=on&FIRST_QUERY_NUM=0&FORMAT_OBJECT=Alignment&FORMAT_PAGE_TARGET=&FORMAT_TYPE=HTML&GET_SEQUENCE=yes&I_THRESH=&MASK_CHAR=2&MASK_COLOR=1&NCBI_GI=yes&NUM_OVERVIEW=100&OLD_BLAST=false&PAGE=MegaBlast&QUERY_INDEX=0&QUERY_NUMBER=0&RESULTS_PAGE_TARGET=&RID=EVX6WZ9X014&SHOW_LINKOUT=yes&SHOW_OVERVIEW=yes&STEP_NUMBER=&OLD_VIEW=false&DISPLAY_SORT=2&HSP_SORT=1) | [**Query cover**](http://www.ncbi.nlm.nih.gov/blast/Blast.cgi?CMD=Get&ALIGNMENTS=0&ALIGNMENT_VIEW=Pairwise&DATABASE_SORT=0&DESCRIPTIONS=100&DYNAMIC_FORMAT=on&FIRST_QUERY_NUM=0&FORMAT_OBJECT=Alignment&FORMAT_PAGE_TARGET=&FORMAT_TYPE=HTML&GET_SEQUENCE=yes&I_THRESH=&MASK_CHAR=2&MASK_COLOR=1&NCBI_GI=yes&NUM_OVERVIEW=100&OLD_BLAST=false&PAGE=MegaBlast&QUERY_INDEX=0&QUERY_NUMBER=0&RESULTS_PAGE_TARGET=&RID=EVX6WZ9X014&SHOW_LINKOUT=yes&SHOW_OVERVIEW=yes&STEP_NUMBER=&OLD_VIEW=false&DISPLAY_SORT=4&HSP_SORT=0) | [**E value**](http://www.ncbi.nlm.nih.gov/blast/Blast.cgi?CMD=Get&ALIGNMENTS=0&ALIGNMENT_VIEW=Pairwise&DATABASE_SORT=0&DESCRIPTIONS=100&DYNAMIC_FORMAT=on&FIRST_QUERY_NUM=0&FORMAT_OBJECT=Alignment&FORMAT_PAGE_TARGET=&FORMAT_TYPE=HTML&GET_SEQUENCE=yes&I_THRESH=&MASK_CHAR=2&MASK_COLOR=1&NCBI_GI=yes&NUM_OVERVIEW=100&OLD_BLAST=false&PAGE=MegaBlast&QUERY_INDEX=0&QUERY_NUMBER=0&RESULTS_PAGE_TARGET=&RID=EVX6WZ9X014&SHOW_LINKOUT=yes&SHOW_OVERVIEW=yes&STEP_NUMBER=&OLD_VIEW=false&DISPLAY_SORT=0&HSP_SORT=0) | [**Identity (%)**](http://www.ncbi.nlm.nih.gov/blast/Blast.cgi?CMD=Get&ALIGNMENTS=0&ALIGNMENT_VIEW=Pairwise&DATABASE_SORT=0&DESCRIPTIONS=100&DYNAMIC_FORMAT=on&FIRST_QUERY_NUM=0&FORMAT_OBJECT=Alignment&FORMAT_PAGE_TARGET=&FORMAT_TYPE=HTML&GET_SEQUENCE=yes&I_THRESH=&MASK_CHAR=2&MASK_COLOR=1&NCBI_GI=yes&NUM_OVERVIEW=100&OLD_BLAST=false&PAGE=MegaBlast&QUERY_INDEX=0&QUERY_NUMBER=0&RESULTS_PAGE_TARGET=&RID=EVX6WZ9X014&SHOW_LINKOUT=yes&SHOW_OVERVIEW=yes&STEP_NUMBER=&DISPLAY_SORT=3&HSP_SORT=3) | **GenBank acession number** |
| --- | --- | --- | --- | --- | --- | --- | --- | --- |
|  |  |  |  |  |  |  |  |  |
| *Thrichomys inermis* | Curaçá/Bahia | [*Leishmania* (*Viannia*) *shawi*](http://www.ncbi.nlm.nih.gov/blast/Blast.cgi#alnHdr_289066227) | 422 | 422 | 100% | 7,00E-115 | 99% | [gi\|289066227\|GU071177.1](http://www.ncbi.nlm.nih.gov/nucleotide/289066227?report=genbank&log$=nucltop&blast_rank=1&RID=EVX6WZ9X014) |
|  |  | [*Leishmania* (*Viannia*) *shawi*](http://www.ncbi.nlm.nih.gov/blast/Blast.cgi#alnHdr_289066227) | 416 | 416 | 100% | 3,00E-113 | 99% | [gi\|289066224\|GU071175.1](http://www.ncbi.nlm.nih.gov/nucleotide/289066224?report=genbank&log$=nucltop&blast_rank=2&RID=EVX6WZ9X014) |
|  |  | [*Leishmania* (*Viannia*) *panamensis*](http://www.ncbi.nlm.nih.gov/blast/Blast.cgi#alnHdr_239586191) | 416 | 416 | 100% | 3,00E-113 | 99% | [gi\|239586191\|EU599094.1](http://www.ncbi.nlm.nih.gov/nucleotide/239586191?report=genbank&log$=nucltop&blast_rank=3&RID=EVX6WZ9X014) |
|  |  | [*Leishmania* (*Viannia*) *panamensis*](http://www.ncbi.nlm.nih.gov/blast/Blast.cgi#alnHdr_239586191) | 416 | 416 | 100% | 3,00E-113 | 99% | [gi\|281426653\|FN395055.1](http://www.ncbi.nlm.nih.gov/nucleotide/281426653?report=genbank&log$=nucltop&blast_rank=4&RID=EVX6WZ9X014) |
|  |  |  |  |  |  |  |  |  |
| *Thrichomys fosteri* | Corumbá/MS | [*Leishmania (Viannia) naiffi*](http://www.ncbi.nlm.nih.gov/blast/Blast.cgi#alnHdr_289066237) | 390 | 390 | 99% | 2,00E-105 | 99% | [gi\|289066237\|GU071183.1](http://www.ncbi.nlm.nih.gov/nucleotide/289066237?report=genbank&log$=nucltop&blast_rank=1&RID=EW1A5HGU015) |
|  |  | [*Leishmania (Viannia) naiffi*](http://www.ncbi.nlm.nih.gov/blast/Blast.cgi#alnHdr_386363692) | 387 | 387 | 99% | 2,00E-104 | 99% | [gi\|386363692\|FR872767.1](http://www.ncbi.nlm.nih.gov/nucleotide/386363692?report=genbank&log$=nucltop&blast_rank=2&RID=EW1A5HGU015) |
|  |  | [*Leishmania (Viannia) naiffi*](http://www.ncbi.nlm.nih.gov/blast/Blast.cgi#alnHdr_289066239) | 385 | 385 | 99% | 9,00E-104 | 99% | [gi\|289066239\|GU071185.1](http://www.ncbi.nlm.nih.gov/nucleotide/289066239?report=genbank&log$=nucltop&blast_rank=3&RID=EW1A5HGU015) |
|  |  | [*Leishmania (Viannia) naiffi*](http://www.ncbi.nlm.nih.gov/blast/Blast.cgi#alnHdr_336087912) | 383 | 383 | 99% | 3,00E-103 | 98% | [gi\|336087912\|FN395056.2](http://www.ncbi.nlm.nih.gov/nucleotide/336087912?report=genbank&log$=nucltop&blast_rank=4&RID=EW1A5HGU015) |
|  |  |  |  |  |  |  |  |  |
| *Dasyprocta azarae* | Aquidauana/MS | [*Leishmania (Leishmania) donovani*](http://www.ncbi.nlm.nih.gov/blast/Blast.cgi#alnHdr_442569722) | 185 | 185 | 98% | 7,00E-44 | 88% | [gi\|442569722\|JX970996.1](http://www.ncbi.nlm.nih.gov/nucleotide/442569722?report=genbank&log$=nucltop&blast_rank=1&RID=G4R776WN013) |
|  |  | [*Leishmania (Leishmania) donovani*](http://www.ncbi.nlm.nih.gov/blast/Blast.cgi#alnHdr_442569720) | 185 | 185 | 98% | 7,00E-44 | 88% | [gi\|442569720\|JX970995.1](http://www.ncbi.nlm.nih.gov/nucleotide/442569720?report=genbank&log$=nucltop&blast_rank=2&RID=G4R776WN013) |
|  |  | [*Leishmania (Leishmania) donovani*](http://www.ncbi.nlm.nih.gov/blast/Blast.cgi#alnHdr_442569718) | 185 | 185 | 98% | 7,00E-44 | 88% | [gi\|442569718\|JX970994.1](http://www.ncbi.nlm.nih.gov/nucleotide/442569718?report=genbank&log$=nucltop&blast_rank=3&RID=G4R776WN013) |
|  |  | [*Leishmania (Leishmania) donovani*](http://www.ncbi.nlm.nih.gov/blast/Blast.cgi#alnHdr_442569716) | 185 | 185 | 98% | 7,00E-44 | 88% | [gi\|442569716\|JX970993.1](http://www.ncbi.nlm.nih.gov/nucleotide/442569716?report=genbank&log$=nucltop&blast_rank=4&RID=G4R776WN013) |
|  |  |  |  |  |  |  |  |  |
| *Clyomys laticeps* | Corumbá/MS | *Leishmania* (*Leishmania*) *donovani* | 427 | 427 | 99% | 2,00E-116 | 99% | [gi\|398018456\|XM_003862348.1](http://www.ncbi.nlm.nih.gov/nucleotide/398018456?report=genbank&log$=nucltop&blast_rank=1&RID=EVXZTMPV014) |
|  |  | *Leishmania* (*Leishmania*) *donovani* | 427 | 427 | 99% | 2,00E-116 | 99% | [gi\|389618739\|JQ990221.1](http://www.ncbi.nlm.nih.gov/nucleotide/389618739?report=genbank&log$=nucltop&blast_rank=2&RID=EVXZTMPV014) |
|  |  | *Leishmania* (*Leishmania*) *donovani* | 427 | 427 | 99% | 2,00E-116 | 99% | [gi\|388850721\|JX021437.1](http://www.ncbi.nlm.nih.gov/nucleotide/388850721?report=genbank&log$=nucltop&blast_rank=3&RID=EVXZTMPV014) |
|  |  | *Leishmania* (*Leishmania*) *donovani* | 427 | 427 | 99% | 2,00E-116 | 99% | gi\|388850707\|JX021430.1 |
|  |  |  |  |  |  |  |  |  |
| *Clyomys laticeps* | Corumbá/MS | *Leishmania* (*Leishmania*) *donovani* | 361 | 361 | 99% | 2,00E-96 | 93% | [gi\|398018456\|XM_003862348.1](http://www.ncbi.nlm.nih.gov/nucleotide/398018456?report=genbank&log$=nucltop&blast_rank=1&RID=EW1226W2014) |
|  |  | *Leishmania* (*Leishmania*) *donovani* | 361 | 361 | 99% | 2,00E-96 | 93% | [gi\|389618739\|JQ990221.1](http://www.ncbi.nlm.nih.gov/nucleotide/389618739?report=genbank&log$=nucltop&blast_rank=2&RID=EW1226W2014) |
|  |  | *Leishmania* (*Leishmania*) *donovani* | 361 | 361 | 99% | 2,00E-96 | 93% | [gi\|388850721\|JX021437.1](http://www.ncbi.nlm.nih.gov/nucleotide/388850721?report=genbank&log$=nucltop&blast_rank=3&RID=EW1226W2014) |
|  |  | *Leishmania* (*Leishmania*) *donovani* | 361 | 361 | 99% | 2,00E-96 | 93% | [gi\|388850707\|JX021430.1](http://www.ncbi.nlm.nih.gov/nucleotide/388850707?report=genbank&log$=nucltop&blast_rank=4&RID=EW1226W2014) |
|  |  |  |  |  |  |  |  |  |
| *Thrichomys fosteri* | Corumbá/MS | [*Leishmania (Viannia) naiffi*](http://www.ncbi.nlm.nih.gov/blast/Blast.cgi#alnHdr_289066239) | 435 | 435 | 99% | 9,00E-119 | 100% | [gi\|289066239\|GU071185.1](http://www.ncbi.nlm.nih.gov/nucleotide/289066239?report=genbank&log$=nucltop&blast_rank=1&RID=EW0SC8VF015) |
|  |  | [*Leishmania (Viannia) naiffi*](http://www.ncbi.nlm.nih.gov/blast/Blast.cgi#alnHdr_386363692) | 431 | 431 | 99% | 1,00E-117 | 99% | [gi\|386363692\|FR872767.1](http://www.ncbi.nlm.nih.gov/nucleotide/386363692?report=genbank&log$=nucltop&blast_rank=2&RID=EW0SC8VF015) |
|  |  | [*Leishmania (Viannia) naiffi*](http://www.ncbi.nlm.nih.gov/blast/Blast.cgi#alnHdr_289066237) | 429 | 429 | 99% | 4,00E-117 | 99% | [gi\|289066237\|GU071183.1](http://www.ncbi.nlm.nih.gov/nucleotide/289066237?report=genbank&log$=nucltop&blast_rank=3&RID=EW0SC8VF015) |
|  |  | [*Leishmania (Viannia) braziliensis*](http://www.ncbi.nlm.nih.gov/blast/Blast.cgi#alnHdr_386363684) | 424 | 424 | 99% | 2,00E-115 | 99% | [gi\|386363684\|FR872763.1](http://www.ncbi.nlm.nih.gov/nucleotide/386363684?report=genbank&log$=nucltop&blast_rank=4&RID=EW0SC8VF015) |
|  |  |  |  |  |  |  |  |  |
| *Thrichomys laurentius* | S. R. Nonato/PI | [*Leishmania (Viannia) guyanensis*](http://www.ncbi.nlm.nih.gov/nucleotide/316891126?report=genbank&log$=nucltop&blast_rank=4&RID=G4TRCD4U015) | 433 | 433 | 99% | 3,00E-118 | 100% | [gi\|316891132\|GU368240.1](http://www.ncbi.nlm.nih.gov/blast/Blast.cgi#alnHdr_316891126) |
|  |  | [*Leishmania (Viannia) guyanensis*](http://blast.ncbi.nlm.nih.gov/Blast.cgi#alnHdr_289066231) | 433 | 433 | 99% | 3,00E-118 | 100% | [gi\|316891130\|GU368239.1](http://blast.ncbi.nlm.nih.gov/Blast.cgi#alnHdr_289066226) |
|  |  | [*Leishmania (Viannia) guyanensis*](http://www.ncbi.nlm.nih.gov/nucleotide/289066236?report=genbank&log$=nucltop&blast_rank=3&RID=GTXZG3TH013) | 433 | 433 | 99% | 3,00E-118 | 100% | [gi\|316891128\|GU368238.1](http://www.ncbi.nlm.nih.gov/nucleotide/289066226?report=genbank&log$=nucltop&blast_rank=1&RID=GTXZG3TH013) |
|  |  | [*Leishmania (Viannia) guyanensis*](http://blast.ncbi.nlm.nih.gov/Blast.cgi#alnHdr_289066236) | 433 | 433 | 99% | 3,00E-118 | 100% | [gi\|316891126\|GU368237.1](http://www.ncbi.nlm.nih.gov/nucleotide/289066231?report=genbank&log$=nucltop&blast_rank=4&RID=GTXZG3TH013) |
|  |  |  |  |  |  |  |  |  |
| *Thrichomys laurentius* | S. R. Nonato/PI | [*Leishmania (Leishmania) donovani*](http://www.ncbi.nlm.nih.gov/blast/Blast.cgi#alnHdr_442569724) | 303 | 303 | 86% | 2,00E-79 | 97% | [gi\|442569724\|JX970997.1](http://www.ncbi.nlm.nih.gov/nucleotide/442569724?report=genbank&log$=nucltop&blast_rank=1&RID=G4T8U70Z013) |
|  |  | [*Leishmania (Leishmania) donovani*](http://www.ncbi.nlm.nih.gov/blast/Blast.cgi#alnHdr_442569722) | 303 | 303 | 86% | 2,00E-79 | 97% | [gi\|442569722\|JX970996.1](http://www.ncbi.nlm.nih.gov/nucleotide/442569722?report=genbank&log$=nucltop&blast_rank=2&RID=G4T8U70Z013) |
|  |  | [*Leishmania (Leishmania) donovani*](http://www.ncbi.nlm.nih.gov/blast/Blast.cgi#alnHdr_442569720) | 303 | 303 | 86% | 2,00E-79 | 97% | [gi\|442569720\|JX970995.1](http://www.ncbi.nlm.nih.gov/nucleotide/442569720?report=genbank&log$=nucltop&blast_rank=3&RID=G4T8U70Z013) |
|  |  | [*Leishmania (Leishmania) donovani*](http://www.ncbi.nlm.nih.gov/blast/Blast.cgi#alnHdr_442569718) | 303 | 303 | 86% | 2,00E-79 | 97% | [gi\|442569718\|JX970994.1](http://www.ncbi.nlm.nih.gov/nucleotide/442569718?report=genbank&log$=nucltop&blast_rank=4&RID=G4T8U70Z013) |
|  |  |  |  |  |  |  |  |  |
| *Thrichomys laurentius* | S. R. Nonato/PI | [*Leishmania* (*Viannia*) *braziliensis*](http://www.ncbi.nlm.nih.gov/blast/Blast.cgi#alnHdr_386363684) | 422 | 422 | 99% | 7,00E-115 | 99% | [gi\|386363684\|FR872763.1](http://www.ncbi.nlm.nih.gov/nucleotide/386363684?report=genbank&log$=nucltop&blast_rank=1&RID=EVYGMYCF01R) |
|  |  | [*Leishmania* (*Viannia*) *naiffi*](http://www.ncbi.nlm.nih.gov/blast/Blast.cgi#alnHdr_289066239) | 422 | 422 | 99% | 7,00E-115 | 99% | [gi\|289066239\|GU071185.1](http://www.ncbi.nlm.nih.gov/nucleotide/289066239?report=genbank&log$=nucltop&blast_rank=2&RID=EVYGMYCF01R) |
|  |  | [*Leishmania* (*Viannia*) *braziliensis*](http://www.ncbi.nlm.nih.gov/blast/Blast.cgi#alnHdr_386363678) | 420 | 420 | 99% | 3,00E-114 | 99% | [gi\|386363678\|FR872760.1](http://www.ncbi.nlm.nih.gov/nucleotide/386363678?report=genbank&log$=nucltop&blast_rank=3&RID=EVYGMYCF01R) |
|  |  | [*Leishmania* (*Viannia*) *braziliensis*](http://www.ncbi.nlm.nih.gov/blast/Blast.cgi#alnHdr_386363680) | 420 | 420 | 99% | 3,00E-114 | 99% | [gi\|386363680\|FR872761.1](http://www.ncbi.nlm.nih.gov/nucleotide/386363680?report=genbank&log$=nucltop&blast_rank=4&RID=EVYGMYCF01R) |
|  |  |  |  |  |  |  |  |  |
| *Thrichomys laurentius* | S. R. Nonato/PI | [*Leishmania* (*Viannia*) *shawi*](http://www.ncbi.nlm.nih.gov/blast/Blast.cgi#alnHdr_289066227) | 390 | 390 | 100% | 2,00E-105 | 95% | [gi\|289066227\|GU071177.1](http://www.ncbi.nlm.nih.gov/nucleotide/289066227?report=genbank&log$=nucltop&blast_rank=1&RID=EVZ38KXX015) |
|  |  | [*Leishmania* (*Viannia*) *guyanensis*](http://www.ncbi.nlm.nih.gov/blast/Blast.cgi#alnHdr_316891132) | 385 | 385 | 100% | 1,00E-103 | 95% | [gi\|316891132\|GU368240.1](http://www.ncbi.nlm.nih.gov/nucleotide/316891132?report=genbank&log$=nucltop&blast_rank=2&RID=EVZ38KXX015) |
|  |  | [*Leishmania* (*Viannia*) *guyanensis*](http://www.ncbi.nlm.nih.gov/blast/Blast.cgi#alnHdr_316891132) | 385 | 385 | 100% | 1,00E-103 | 95% | [gi\|316891130\|GU368239.1](http://www.ncbi.nlm.nih.gov/nucleotide/316891130?report=genbank&log$=nucltop&blast_rank=3&RID=EVZ38KXX015) |
|  |  | [*Leishmania* (*Viannia*) *guyanensis*](http://www.ncbi.nlm.nih.gov/blast/Blast.cgi#alnHdr_316891132) | 385 | 385 | 100% | 1,00E-103 | 95% | [gi\|316891128\|GU368238.1](http://www.ncbi.nlm.nih.gov/nucleotide/316891128?report=genbank&log$=nucltop&blast_rank=4&RID=EVZ38KXX015) |
|  |  |  |  |  |  |  |  |  |
| *Thrichomys laurentius* | S. R. Nonato/PI | [*Leishmania (Viannia) naiffi*](http://www.ncbi.nlm.nih.gov/blast/Blast.cgi#alnHdr_289066239) | 435 | 435 | 100% | 9,00E-119 | 100% | [gi\|289066239\|GU071185.1](http://www.ncbi.nlm.nih.gov/nucleotide/289066239?report=genbank&log$=nucltop&blast_rank=1&RID=EW07TG08014) |
|  |  | [*Leishmania (Viannia) naiffi*](http://www.ncbi.nlm.nih.gov/blast/Blast.cgi#alnHdr_386363692) | 431 | 431 | 100% | 1,00E-117 | 99% | [gi\|386363692\|FR872767.1](http://www.ncbi.nlm.nih.gov/nucleotide/386363692?report=genbank&log$=nucltop&blast_rank=2&RID=EW07TG08014) |
|  |  | [*Leishmania (Viannia) naiffi*](http://www.ncbi.nlm.nih.gov/blast/Blast.cgi#alnHdr_289066237) | 429 | 429 | 100% | 4,00E-117 | 99% | [gi\|289066237\|GU071183.1](http://www.ncbi.nlm.nih.gov/nucleotide/289066237?report=genbank&log$=nucltop&blast_rank=3&RID=EW07TG08014) |
|  |  | [*Leishmania (Viannia) braziliensis*](http://www.ncbi.nlm.nih.gov/blast/Blast.cgi#alnHdr_386363684) | 424 | 424 | 100% | 2,00E-115 | 99% | [gi\|386363684\|FR872763.1](http://www.ncbi.nlm.nih.gov/nucleotide/386363684?report=genbank&log$=nucltop&blast_rank=4&RID=EW07TG08014) |
|  |  |  |  |  |  |  |  |  |
| *Thrichomys laurentius* | S. R. Nonato/PI | [*Leishmania* (*Viannia*) *naiffi*](http://www.ncbi.nlm.nih.gov/blast/Blast.cgi#alnHdr_289066239) | 422 | 422 | 100% | 7,00E-115 | 99% | [gi\|289066239\|GU071185.1](http://www.ncbi.nlm.nih.gov/nucleotide/289066239?report=genbank&log$=nucltop&blast_rank=1&RID=EW0FXKV7014) |
|  |  | [*Leishmania* (*Viannia*) *naiffi*](http://www.ncbi.nlm.nih.gov/blast/Blast.cgi#alnHdr_386363692) | 418 | 418 | 100% | 9,00E-114 | 99% | [gi\|386363692\|FR872767.1](http://www.ncbi.nlm.nih.gov/nucleotide/386363692?report=genbank&log$=nucltop&blast_rank=2&RID=EW0FXKV7014) |
|  |  | [*Leishmania* (*Viannia*) *naiffi*](http://www.ncbi.nlm.nih.gov/blast/Blast.cgi#alnHdr_289066237) | 416 | 416 | 100% | 3,00E-113 | 99% | [gi\|289066237\|GU071183.1](http://www.ncbi.nlm.nih.gov/nucleotide/289066237?report=genbank&log$=nucltop&blast_rank=3&RID=EW0FXKV7014) |
|  |  | [*Leishmania* (*Viannia*) *braziliensis*](http://www.ncbi.nlm.nih.gov/blast/Blast.cgi#alnHdr_386363684) | 411 | 411 | 100% | 2,00E-111 | 98% | [gi\|386363684\|FR872763.1](http://www.ncbi.nlm.nih.gov/nucleotide/386363684?report=genbank&log$=nucltop&blast_rank=4&RID=EW0FXKV7014) |
|  |  |  |  |  |  |  |  |  |
| *Thrichomys laurentius ** | S. R. Nonato/PI | *Leishmania* (*Viannia*) *guyanensis* | 435 | 435 | 99% | 1,00E-118 | 100% | [gi\|316891079\|GU368213.1](http://www.ncbi.nlm.nih.gov/nucleotide/316891079?report=genbank&log$=nucltop&blast_rank=1&RID=KZN758P6014) |
|  |  | *Leishmania* (*Viannia*) *guyanensis* | 435 | 435 | 99% | 1,00E-118 | 100% | [gi\|316891075\|GU368211.1](http://www.ncbi.nlm.nih.gov/nucleotide/316891075?report=genbank&log$=nucltop&blast_rank=2&RID=KZN758P6014) |
|  |  | *Leishmania* (*Viannia*) *guyanensis* | \| 435 \| \| --- \| | 435 | 99% | 1,00E-118 | 100% | [gi\|316891077\|GU368212.1](http://www.ncbi.nlm.nih.gov/nucleotide/316891077?report=genbank&log$=nucltop&blast_rank=3&RID=KZN758P6014) |
|  |  | *Leishmania* (*Viannia*) *guyanensis* | 435 | 435 | 99% | 1,00E-118 | 100% | [gi\|289066218\|GU071172.1](http://www.ncbi.nlm.nih.gov/nucleotide/289066218?report=genbank&log$=nucltop&blast_rank=4&RID=KZN758P6014) |
|  |  |  |  |  |  |  |  |  |
| *Thrichomys laurentius ** | S. R. Nonato/PI | [*Leishmania* (*Viannia*) *braziliensis*](http://www.ncbi.nlm.nih.gov/blast/Blast.cgi#alnHdr_386363684) | 416 | 416 | 99% | 3,00E-113 | 99% | [gi\|386363684\|FR872763.1](http://www.ncbi.nlm.nih.gov/nucleotide/386363684?report=genbank&log$=nucltop&blast_rank=1&RID=EVZVSEP3015) |
|  |  | [*Leishmania* (*Viannia*) *guyanensis*](http://www.ncbi.nlm.nih.gov/blast/Blast.cgi#alnHdr_316891132) | 416 | 416 | 99% | 3,00E-113 | 99% | [gi\|316891132\|GU368240.1](http://www.ncbi.nlm.nih.gov/nucleotide/316891132?report=genbank&log$=nucltop&blast_rank=2&RID=EVZVSEP3015) |
|  |  | [*Leishmania* (*Viannia*) *guyanensis*](http://www.ncbi.nlm.nih.gov/blast/Blast.cgi#alnHdr_316891130) | 416 | 416 | 99% | 3,00E-113 | 99% | [gi\|316891130\|GU368239.1](http://www.ncbi.nlm.nih.gov/nucleotide/316891130?report=genbank&log$=nucltop&blast_rank=3&RID=EVZVSEP3015) |
|  |  | [*Leishmania* (*Viannia*) *guyanensis*](http://www.ncbi.nlm.nih.gov/blast/Blast.cgi#alnHdr_316891128) | 416 | 416 | 99% | 3,00E-113 | 99% | [gi\|316891128\|GU368238.1](http://www.ncbi.nlm.nih.gov/nucleotide/316891128?report=genbank&log$=nucltop&blast_rank=4&RID=EVZVSEP3015) |

* Analysis of the DNA sequencing obtained in distinct PCR reactions with the same rodent sample.
